# Marburg Heart Score and INTERCHEST score for telephone triage of acute chest pain: a prospective, diagnostic accuracy study in out-of-hours primary care

**DOI:** 10.1136/bmjopen-2025-111113

**Published:** 2026-04-07

**Authors:** Amy Manten, Indra M B Melessen, Jelle C L Himmelreich, Eric P Moll van Charante, Ralf E Harskamp

**Affiliations:** 1 Department of General Practice, Amsterdam University Medical Centres, University of Amsterdam, Amsterdam, Netherlands; 2 Atherosclerosis & Ischemic Syndromes, Amsterdam Cardiovascular Sciences, Amsterdam, Netherlands; 3 Department of Public and Occupational Health, Amsterdam University Medical Centres, University of Amsterdam, Amsterdam, Netherlands

**Keywords:** Primary Health Care, General Practice, Myocardial infarction, Ischaemic heart disease, Cardiovascular Disease, Triage

## Abstract

**Objectives:**

To assess whether the Marburg Heart Score (MHS) and INTERCHEST score may improve telephone triage of chest pain by providing better diagnostic discrimination compared with the triage protocol from the Netherlands Triage Standard (NTS).

**Design:**

Prospective diagnostic accuracy study.

**Setting:**

Large regional out-of-hours primary care (OOH-PC) facility in Alkmaar, the Netherlands.

**Participants:**

A total of 1254 eligible patients contacted the OOH-PC facility (median age 56.0 years, 57.9% female) between December 2022 and May 2023. The study was completed and verbal informed consent obtained in 280 (22.3%) patients.

**Interventions:**

Triage assistants asked study questions in addition to the NTS protocol to complete the MHS and INTERCHEST score.

**Primary and secondary outcome measures:**

Discrimination (C-statistics) and diagnostic test properties (eg, sensitivity/specificity) were used; the reference standard was the occurrence of a major event (ie, composite of all-cause mortality, and urgent cardiovascular and non-cardiovascular conditions) or acute coronary syndrome (ACS) within 6 weeks.

**Results:**

A major event occurred in 36 patients (12.9%), including 13 (4.6%) ACS cases. For predicting major events, the MHS and INTERCHEST scores showed C-statistics of 0.67 (95% CI 0.57 to 0.77) and 0.64 (95% CI 0.54 to 0.74), respectively, compared with 0.62 (95% CI 0.53 to 0.71) for the NTS protocol. For ACS, C-statistics were 0.62 (95% CI 0.45 to 0.79), 0.59 (95% CI 0.43 to 0.75), and 0.62 (95% CI 0.49 to 0.75) for MHS, INTERCHEST and NTS, respectively. Regarding test characteristics, the MHS and INTERCHEST score showed higher point estimates for specificity (27.9% and 26.6%) vs the NTS (19.7%), but at the expense of lower sensitivity (88.9% and 86.1% versus 97.2%) for major events. For ACS, a similar pattern was observed (specificity 26.2% and 25.5% vs 18.4; sensitivity 84.6% and 84.6% vs 100.0%).

**Conclusions:**

Simple clinical decision rules (MHS and INTERCHEST) have comparable, modest discriminative ability and diagnostic properties compared with the current protocol for telephone triage of acute chest pain in Dutch OOH-PC.

**Trial registration number:**

Netherlands Trial Register (TRACE – NL-OMON20102).

**STRENGTHS AND LIMITATIONS OF THIS STUDY**

- First head-to-head, prospective comparison of the performance of the MHS and INTERCHEST score with the current standard for telephone triage.
- Broad outcome definition using major events to capture the broad range of serious underlying causes of chest pain.
- Possible selection bias as study questions were completed in only 22.3% of eligible patients.
- Patients did not undergo a standardised diagnostic work-up; possible verification bias was countered by a prolonged follow-up.

## Introduction

 Acute chest pain presents a significant challenge in prehospital settings, with causes ranging from benign musculoskeletal complaints to urgent aetiologies such as acute coronary syndrome (ACS), pulmonary embolism or aortic dissection.^1^,^4^ Rapid identification of patients requiring urgent care is crucial to enable timely treatment and prevent complications. However, prehospital settings lack the extensive diagnostic abilities offered by hospitals.

In the Netherlands, out-of-hours primary care (OOH-PC) facilities play a key role in prehospital care, managing approximately 11 chest pain-related contacts per 1000 inhabitants each year.^5^,^7^ The facilities function as an extension of general practitioners’ position as gatekeepers, helping to coordinate and facilitate safe access to secondary care. Initial assessments are typically conducted over the telephone. In 2011, protocols from the Netherlands Triage Standard (NTS) were introduced to support timely identification of urgent cases and reduce unnecessary diagnostic testing or treatment.^8^ The NTS protocol for chest pain consists of an assessment of the haemodynamic status and is followed by seven hierarchically structured questions to estimate the urgency. However, studies have reported disappointing diagnostic accuracies, with frequent overestimations and underestimations.^9 10^

The Marburg Heart Score (MHS) and INTERCHEST score are clinical decision rules developed to rule out coronary artery disease (CAD) in daytime primary care practices.^11 12^ The MHS, developed in 2010, consists of five elements: age/sex criteria, known cardiovascular disease, exercise-related pain, reproducibility and whether the patient assumes a cardiac cause.^12^ The INTERCHEST score, developed in 2017, incorporates six items that are similar to those of the MHS plus physician suspicion and a pressure-type sensation.^11^ Both scores have shown promise in primary care settings with C-statistics ranging from 0.64 to 0.85 for predicting ACS or major adverse cardiovascular events.^13^ Retrospective evaluation of the scores as triage tools demonstrated potential advantages over the NTS protocol.^14^

In this prospective study, we evaluated the discriminatory and diagnostic test properties of the MHS and INTERCHEST score for ACS or other major events in patients with chest pain contacting an OOH-PC facility in the Netherlands, and compared their performance to the current Dutch triage protocol.

## Methods

We reported our study in accordance with the Standards for Reporting of Diagnostic Accuracy Studies 2015 statement.^15^ The study protocol was reviewed by the Medical Ethical Review Committee of the Amsterdam University Medical Centres, location AMC, and was exempted from full evaluation, as it did not fall within the scope of the Dutch WMO.

### Setting and study design

This study was conducted at a large regional OOH-PC facility in Alkmaar, the Netherlands, serving approximately 250 000 inhabitants with demographic characteristics representative of the overall Dutch population. This study represents the prospective phase of the TRACE project. A detailed description of earlier project phases and an overview of their results has been published previously.^9 16^ All consecutive patients (≥18 years) who contacted the facility with chest pain between 1 December 2022 and 31 May 2023 were eligible for inclusion. On contact, triage assistants used the chest pain-specific NTS protocol to assess urgency. For study purposes, they asked four additional chest pain-related questions along with two additional assessments to complete the MHS and INTERCHEST score elements (online supplement 1). These study items were not integrated into the digital triage system and did not influence urgency codes. Researchers contacted participants at least 6 weeks later to collect follow-up data on patient-reported clinical outcomes. Although the study did not require a formal patient consent, triage assistants and researchers were instructed to obtain verbal consent during both the initial and follow-up contact. For eligible patients who were not asked additional study questions, we only collected baseline information (ie, age, sex, standardised NTS elements), unless they opted out. These patients were not contacted for follow-up, and no identifiable data were available to the research team. Patients who actively declined participation were excluded from all analyses.

### Clinical decision rules

The individual components and scoring rules of the MHS and INTERCHEST scores are outlined in table 1. The MHS consists of five elements: (1) female ≥65 years or male ≥55 years, (2) known CAD, cerebrovascular disease or peripheral vascular disease, (3) pain worsened by exercise, (4) pain reproducible with palpation and (5) the patient assumes a cardiac cause. Each element is scored 0 or 1 points, resulting in a total score ranging from 0 to 5.^12^ The INTERCHEST score is a similar point-based score containing six elements; (1) history of CAD, (2) female ≥65 years or male ≥65 years, (3) chest pain related to effort, (4) pain reproducible with palpation, (5) physician initially suspects a serious condition (adapted to the triage assistant’s sense of alarm, scored as ≥5.5 on 1–10 scale) and (6) chest discomfort described as ‘pressure’. Each element scores 0 or 1 points, except reproducibility, which scores −1 when present, resulting in a total score ranging from −1 to 5.^11^ We extracted all elements from the triage registry, including standardised NTS items and responses to additional study questions. During data coding, symptoms were classified as present when explicitly reported, and otherwise coded as absent. Scores were calculated by a researcher blinded to clinical outcomes.

**Table 1:** Elements of the current triage protocol for chest pain (NTS), the MHS and INTERCHEST score

NTS protocol for chest pain	Marburg heart score	No	Yes	INTERCHEST score	No	Yes
Check of haemodynamic status	Female ≥65 years or male ≥55	0	+1	History of CAD	0	+1
Type of pain	Years			Female ≥65 years or male ≥55	0	+1
Pain duration	Known CAD, cerebrovascular	0	+1	Years		
Severity (1–10 scale)	Disease or peripheral vascular			Chest pain related to effort	0	+1
Course of the pain	disease			Reproducible with palpation	0	−1
Location on the chest	Pain worse with exercise	0	+1	Triage assistant’s sense of alarm	0	+1
Radiation	Reproducible with palpation	+1	0	(≥5.5 on 1–10 scale)		
Associated symptoms	Patient assumes cardiac cause	0	+1	Chest discomfort feels like	0	+1
				‘pressure’		
Results in urgency code (U1–U5)	Results in score 0–5					
				Results in score −1 to 5		

CAD, coronary artery disease; MHS, Marburg Heart Score; NTS, Netherlands Triage Standard.

### Netherlands Triage Standard

During the study period, the OOH-PC facility employed version 9.50 of the NTS chest pain protocol.^8^ The protocol initiates with a haemodynamic assessment, followed by seven hierarchical chest pain-specific questions that address pain characteristics, location, radiation and associated symptoms (table 1). Based on the responses, the NTS generates urgency codes (U1–U5) that correspond to a recommended time-to-care. U1 and U2 are considered high urgencies requiring care within 60 min. Possible triage outcomes include ambulance dispatch, a general practitioner (GP) home visit or on-site consultation, or telephone advice. Triage assistants retain the ability to deviate from recommended urgency and/or action after consulting the attending GP.

### Clinical outcomes of interest

We assessed the occurrence of a major event or ACS within 6 weeks after the index contact. Major events were defined as a composite of all-cause mortality, and urgent cardiovascular and non-cardiovascular conditions, linked to the initial chest pain complaint and requiring hospital admission and/or urgent in-hospital treatment (online supplement 2). We verified all patient-reported major events and gathered additional details through contact with the patient’s GP, using supporting hospitalisation and discharge letters related to the index consultation.

### Analysis

We described patient characteristics (eg, age, sex) and triage characteristics (eg, time of contact, symptom features based on NTS elements, assigned urgency and action following triage) for all eligible patients. Patients with available data on additional study questions and data on clinical outcomes were included in performance analyses of the MHS, INTERCHEST score and current triage protocol (NTS). Diagnostic accuracy measures (sensitivity, specificity, positive and negative predictive values (NPV)) were calculated using the presence or absence of a major event, and presence or absence of an ACS, respectively. We compared the performance of the MHS and INTERCHEST score to that of the NTS. The NTS was deemed positive when it allocated a high urgency code (U1 or U2). For the MHS and INTERCHEST, no established triage cut-offs exist. Therefore, we evaluated several thresholds to identify optimal diagnostic performance. Threshold selection was based on clinical interpretability, seeking the most favourable balance between sensitivity and specificity. We reported false positive rates (defined here as the number of false positives divided by the total number of patients in the analysis) to illustrate the percentage of patients that were unnecessarily identified as urgent by each test and respective cut-off.

We assessed the ability to discriminate between patients with and without a major event or ACS, using C-statistics. We visualised discrimination results using receiver operating characteristic (ROC) curves and corresponding areas under the curve (AUC). ROC curves were constructed, and 95% CIs were estimated using the non-parametric approach. No model-based fitting was applied. When the lower bound of the C-statistic’s 95% CI was ≤0.50 we concluded that the model was unable to discriminate for the outcome of interest. For the ROC analyses, NTS urgency levels (U1–U5) were treated as an ordinal predictor, with U1 presenting the highest urgency and U5 the lowest. We performed all statistical analyses using IBM SPSS Statistics (V.28).

After study enrolment was completed, the NTS protocol for chest pain was updated (October 2024). The protocol was revised to include elements of the Safety First model, a symptom-based prediction rule developed in 2022 to rule out ACS in patients with chest pain in OOH-PC.^17^ To enable an up-to-date comparison between the clinical decision rules and the updated protocol, we performed an exploratory analysis of the revised NTS protocol. Online supplement 3 presents the decision tree underlying the update and provides further details on the Safety First model.^18^

### Intended sample size

In a previous retrospective evaluation of the NTS protocol for chest pain by our research group, over 2000 consecutive patients contacted the OOH-PC facility regarding chest pain in 2017. Of these, 88.3% were included, and 16.2% experienced a major event.^9^ Extrapolating these proportions to a planned 6-month inclusion period yields an expected 165 major events if all eligible patients are enrolled. The MHS and INTERCHEST score together introduce seven new predictors for analysis. Applying the conventional rule of 10 events per predictor requires at least 70 events. Therefore, an inclusion rate of ≥42.4% is needed to achieve the minimum number of events for reliable estimation.

### Patient and public involvement

None.

## Results

### Patient characteristics

**Figure 1:**
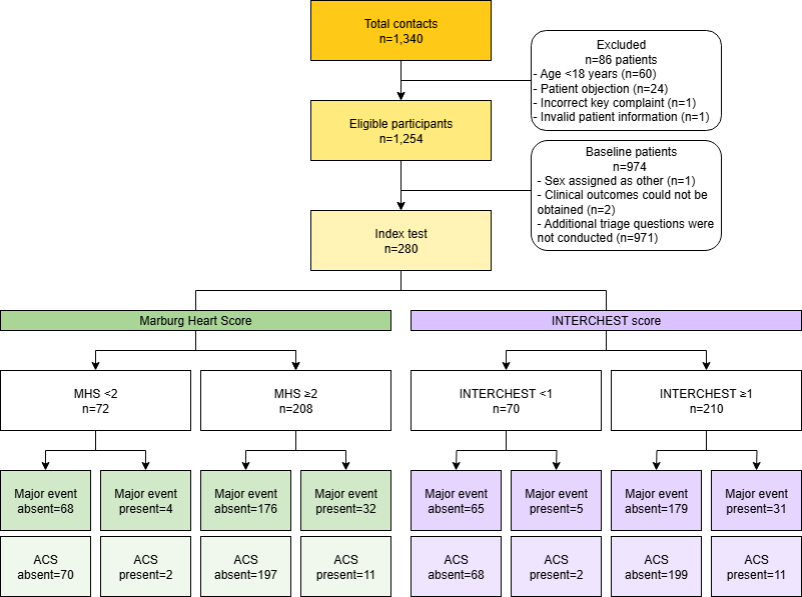
Flow of participants through the study. The figure illustrates the inclusion and exclusion of patients. The MHS and INTERCHEST score are included as index tests. Performance results of the NTS protocol are not included in the flow chart. Major events were defined as a composite of all-cause mortality and urgent conditions linked to the initial chest pain complaint and requiring hospital admission and/or urgent in-hospital treatment. ACS, acute coronary syndrome; MHS, Marburg Heart Score; NTS, Netherlands Triage Standard.

During the inclusion period, 1254 eligible patients contacted the facility with chest pain (median age 56.0 years, 57.9% female). Figure 1 shows patient inclusion and exclusion. High urgency codes (U1 or U2) were assigned to 843 (67.2%) patients. Triage assistants completed the study questions in 280 (22.3%) patients (median age 58.0 years, 57.9% female). Compared with patients without registered study questions, those with available answers showed higher-risk profiles with more typical symptom characteristics and higher urgency. More details are provided in table 2.

**Table 2:** NTS protocol items and triage outcomes among eligible patients

NTS item	Eligible participants (n=1254)	Study questions not conducted (n=974)	Available data on study questions and clinical outcomes (n=280)	P value
Haemodynamic check				
AVPU				
Alert	1250 (99.7)	970 (99.6)	280 (100.0)	0.58
Verbal	1 (0.1)	1 (0.1)	–	1.00
Not registered	3 (0.2)	3 (0.3)	–	1.00
Colour				
Normal	1005 (80.1)	778 (79.9)	227 (81.1)	0.66
Red/flushed	60 (4.8)	46 (4.7)	14 (5.0)	0.85
Pallor	164 (13.1)	133 (13.7)	31 (11.1)	0.26
Ashen	22 (1.8)	14 (1.4)	8 (2.9)	0.11
Not registered	3 (0.2)	3 (0.3)	–	1.00
External bleeding				
No	1243 (99.1)	963 (98.9)	280 (100.0)	0.14
Moderately	3 (0.2)	3 (0.3)	–	1.00
Not registered	8 (0.6)	8 (0.8)	–	0.21
Stridor				
No	1221 (97.4)	946 (97.1)	275 (98.2)	0.32
Non-obstructive	26 (2.1)	21 (2.2)	5 (1.8)	0.70
Yes	2 (0.2)	2 (0.2)	–	1.00
Not registered	5 (0.4)	5 (0.5)	–	0.59
Need for resuscitation				
No	1250 (99.7)	971 (99.7)	279 (99.6)	1.00
Not registered	4 (0.3)	3 (0.3)	1 (0.4)	1.00
Chest pain questions				
Severity*				
Mild (<4)	337 (26.9)	259 (26.6)	78 (27.9)	0.67
Moderate (5-7)	618 (49.3)	469 (48.2)	149 (53.2)	0.14
Severe (8-10)	113 (9.0)	82 (8.4)	31 (11.1)	0.17
No chest pain	169 (13.5)	151 (15.5)	18 (6.4)	<0.001
Not registered	17 (1.4)	13 (1.3)	4 (1.4)	1.00
Duration				
<12 hours	630 (50.2)	482 (49.5)	148 (52.9)	0.32
>12 hours and unchanged	240 (19.1)	189 (19.4)	51 (18.2)	0.66
>12 hours and changed/worsened	197 (15.7)	147 (15.1)	50 (17.9)	0.26
Not registered	187 (14.9)	156 (16.0)	31 (11.1)	0.041
Location				
Left sided	340 (27.1)	275 (28.2)	65 (23.2)	0.10
Right sided	106 (8.5)	98 (10.1)	8 (2.9)	<0.001
Middle of chest	385 (30.7)	281 (28.9)	104 (37.1)	0.008
Band-like around chest	71 (5.7)	48 (4.9)	23 (8.2)	0.036
Not registered	352 (28.1)	272 (27.9)	80 (28.6)	0.83
Radiation				
None	469 (37.4)	404 (41.5)	65 (23.2)	<0.001
Typical radiation†	193 (15.4)	142 (14.6)	51 (18.2)	0.14
Atypical radiation†	202 (16.1)	168 (17.2)	34 (12.1)	0.041
Not registered	390 (31.1)	260 (26.7)	130 (46.4)	<0.001
Type of pain				
Pressure/heavy	227 (18.1)	162 (16.6)	65 (23.2)	0.012
Stabbing/sharp	294 (23.4)	258 (26.5)	36 (12.9)	<0.001
Related to respiration	142 (11.3)	119 (12.2)	23 (8.2)	0.06
Unclear	155 (12.4)	138 (14.2)	17 (6.1)	<0.001
Not registered	436 (34.8)	297 (30.5)	139 (49.6)	<0.001
Course				
Gradual increasing intensity	301 (24.0)	249 (25.6)	52 (18.6)	0.016
Typical and subsided	23 (1.8)	18 (1.8)	5 (1.8)	0.95
Atypical and subsided	62 (4.9)	54 (5.5)	8 (2.9)	0.07
Rapidly progressive	9 (0.7)	7 (0.7)	2 (0.7)	1.00
Not registered	859 (68.5)	646 (66.3)	213 (76.1)	0.002
Accompanying symptoms‡				
Yes	46 (3.7)	35 (3.6)	11 (3.9)	0.79
Subsided	27 (2.2)	23 (2.4)	4 (1.4)	0.34
No	670 (53.4)	565 (58.0)	105 (37.5)	<0.001
Not registered	511 (40.7)	351 (36.0)	160 (57.1)	<0.001
Triage outcomes				
High urgency (U1/U2)	830 (66.2)	604 (62.0)	226 (80.7)	<0.001
Activation of ambulance services	469 (37.4)	314 (32.2)	155 (55.4)	<0.001

The table illustrates the occurrence of symptom characteristics across the standardised NTS questions included in the chest pain protocol and provides the main triage outcomes. Results are shown separately for all eligible patients and for patients with and without available data on additional study questions and clinical outcomes. P value indicates difference between patients with and without available data.

The AVPU score is a four-level assessment tool to determine a patient's level of conciousness and responsiveness, standing for alert, verbal, pain or unresponsive.

* Chest pain severity is scored on a 1–10 scale, 10 being most severe.

† Triage assistants base whether radiation is typical or atypical by assessing the location and character of the radiation (eg, pain, heaviness, tingling sensation).

‡ Possible accompanying symptoms include sweating, nausea, pallor, fear/anxiety and (near) fainting.

§ The AVPU score is a four-level assessment tool to determine a patient's level of conciousness and responsiveness, standing for alert, verbal, pain or unresponsive.

NTS, Netherlands Triage Standard.

### Clinical outcomes

Among the 280 patients with available data on study questions and clinical outcomes, 36 (12.9%) experienced a major event within 6 weeks, including 13 (4.6%) ACS cases (4 ST-elevated myocardial infarction (STEMI), 6 non-STEMI (NSTEMI), 1 myocardial infarction with non-obstructive coronary arteries, 1 unstable angina and 1 unspecified ACS). Most major events (79.5%) were diagnosed on the same day as the index contact. All-cause mortality (<6 weeks) occurred in 3 (1.1%) patients. Among those without a major event, musculoskeletal causes of chest pain were most common (in 39.6%).

### Diagnostic performance

**Figure 2:**
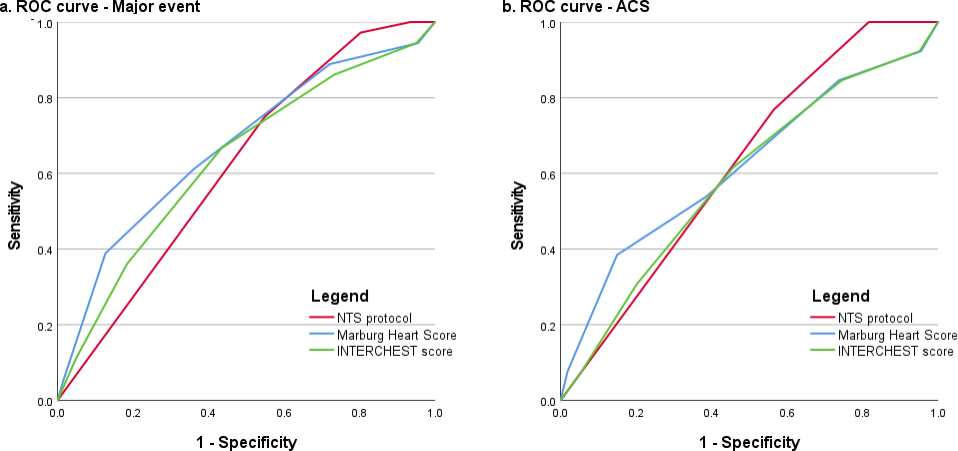
ROC curves illustrating the discriminative ability of the NTS protocol, MHS and INTERCHEST score for predicting (**a**) major events and (**b**) ACS. The curve representing the NTS protocol was based on the allocation of a high urgency code (U1 or U2). The curves for the two clinical decision rules were based on the score results, ranging from 0 to 5 points (MHS) or −1 to 5 points (INTERCHEST). ACS, acute coronary syndrome; MHS, Marburg Heart Score; NTS, Netherlands Triage Standard; ROC, receiver-operating characteristics.

The MHS and INTERCHEST score resulted in C-statistics of 0.67 (95% CI 0.57 to 0.77) and 0.64 (95% CI 0.54 to 0.74) for major events. The NTS protocol demonstrated a C-statistic of 0.62 (95% CI 0.53 to 0.71) for major events. In the current sample, the MHS, INTERCHEST score and NTS were unable to discriminate for ACS with C-statistics of 0.62 (95% CI 0.45 to 0.79), 0.59 (95% CI 0.43 to 0.75) and 0.62 (95% CI 0.49 to 0.75), respectively (figure 2). Table 3 summarises the diagnostic test characteristics of the clinical decision rules and the NTS protocol. For both the MHS and INTERCHEST score, only thresholds with at least similar false-negative rates for major events are shown.

**Table 3:** Diagnostic accuracy of the NTS protocol, MHS and INTERCHEST score for predicting major events and ACS

	Threshold	Positive test	Sensitivity (%)	Specificity (%)	PPV (%)	NPV (%)
Major event						
NTS protocol	U1/U2	82.5%	97.2 (85.5–99.9)	19.7 (14.9–25.2)	15.2 (14.1–16.3)	98.0 (87.2–99.7)
MHS	≥1	95.4%	94.4 (81.3–99.3)	4.5 (2.3–7.9)	12.7 (11.8–13.7)	84.6 (56.0–96.0)
	≥2	74.3%	88.9 (73.9–96.9)	27.9 (22.3–34.0)	15.4 (13.7–17.3)	94.4 (86.9–97.8)
INTERCHEST	≥0	95.0%	94.4 (81.3–99.3)	4.9 (2.6–8.4)	12.8 (11.9–13.8)	85.7 (58.3–96.3)
	≥1	75.0%	86.1 (70.5–95.3)	26.6 (21.2–32.7)	14.8 (13.0–16.8)	92.9 (84.9–96.8)
	≥2	46.4%	66.7 (49.0–81.4)	56.6 (50.1–62.9)	18.5 (14.7–22.9)	92.0 (87.7–94.9)
ACS						
NTS protocol	U1/U2	82.5%	100.0 (75.3–100)	18.4 (13.9–23.5)	5.6 (5.3–5.9)	100.0 (92.8–100)
MHS	≥1	95.4%	92.3 (64.0–99.8)	4.5 (2.3–7.7)	4.5 (3.9–5.2)	92.3 (62.8–98.8)
	≥2	74.3%	84.6 (54.6–98.1)	26.2 (21.0–31.9)	5.3 (4.2–6.6)	97.2 (90.6–99.2)
INTERCHEST	≥0	95.0%	92.3 (64.0–99.8)	4.9 (2.6–8.2)	4.5 (3.9–5.3)	92.9 (64.8–98.9)
	≥1	75.0%	84.6 (54.6–98.1)	25.5 (20.4–31.1)	5.2 (4.2–6.6)	97.1 (90.3–99.2)
	≥2	46.4%	61.5 (31.6–86.1)	54.3 (48.1–60.4)	6.2 (4.0–9.3)	96.7 (93.5–98.3)

The table lists the diagnostic performance of the NTS protocol for chest pain and the two clinical decision rules. For both the MHS and INTERCHEST score, only thresholds with at least similar false-negative rates for major events are shown.

ACS, acute coronary syndrome; MHS, Marburg Heart Score; NPV, negative predictive value; NTS, Netherlands Triage Standard; PPV, positive predictive value.

The MHS showed optimal diagnostic accuracy at a threshold of ≥2 points. At this threshold, it classified 74.3% as high-risk and failed to identify four major events. The false positive rate was lower than that of the NTS (62.9%), resulting in a sensitivity and specificity of 88.9% (95% CI 73.9% to 96.9%) and 27.9% (95% CI 22.3% to 34.0%), respectively. For ACS diagnosis, MHS (threshold ≥2) missed 2 cases and had a false positive rate of 70.4%, yielding a sensitivity and specificity of 84.6% (95% CI 54.6% to 98.1%) and 26.2% (95% CI 21.0% to 31.9%), respectively.

The INTERCHEST score showed optimal diagnostic accuracy at a threshold of ≥1 point. At this threshold, the score classified 75.0% as high-risk, while failing to identify five major events. The false positive rate was lower than that of the NTS (63.9%), resulting in a sensitivity and specificity of 86.1% (95% CI 70.5% to 95.3%) and 26.6% (95% CI 21.2% to 32.7%), respectively. For ACS, the INTERCHEST score (≥1 threshold) missed 2 cases and had a false positive rate of 71.1%, yielding a sensitivity and specificity of 84.6% (95% CI 54.6% to 98.1%) and 25.5% (95% CI 20.4% to 31.1%), respectively.

The NTS protocol assigned high urgencies (U1 or U2) to 231/280 (82.5%) patients, correctly identifying 35/36 patients with a major event. However, 70% of patients were false positive, thus resulting in a sensitivity and specificity of 97.2% (95% CI 85.5% to 99.9%) and 19.7% (95% CI 14.9% to 25.2%), respectively. For ACS, all 13 cases were identified by the NTS (100% sensitivity), but two thirds of patients (77.9%) were false positive, resulting in a specificity of 18.4% (95% CI 13.9% to 23.5%).

### Exploratory analysis: updated NTS protocol

The updated NTS protocol showed C-statistics of 0.59 (95% CI 0.50 to 0.67) for major event prediction and 0.64 (95% CI 0.51 to 0.77) for ACS. It assigned high urgency codes to only 94 (33.6%) patients, resulting in higher specificity (67.2% (95% CI 60.9% to 73.1%) for major events, 67.4% (95% CI 61.4% to 73.0%) for ACS). The updated protocol missed 22/36 (61.1%) major events and 6/13 (46.2%) ACS cases, causing a reduced sensitivity (38.9% (95% CI 23.1% to 56.5%) for major events, 53.9% (95% CI 25.1% to 80.8%) for ACS) (online supplement 4).

## Discussion

In this prospective study, we found that the MHS and INTERCHEST scores had comparable discrimination, with higher specificity but lower sensitivity, compared with the current NTS protocol in patients contacting OOH-PC with acute chest pain. Given that optimal safety is the most important characteristic for triage purposes, this limits the clinical utility of these simple clinical decision rules for telephone triage.

### Strengths and limitations

In this prospective study, we provide a head-to-head comparison between the performance of two clinical decision rules (ie, MHS and INTERCHEST score) and the current standard for triage of acute chest pain in OOH-PC. We also included an exploratory analysis on the updated NTS protocol, implemented in October 2024. By using major events as a primary clinical outcome, we captured a broad range of serious underlying conditions of chest pain. Finally, although our study was conducted in the Netherlands, the findings may apply to other developed countries with similarly structured healthcare systems and integrated triage systems.

Our study also has limitations. First, the target inclusion rate of ≥42.4% was not reached, as triage assistants completed study questions in only 22.3% of eligible patients. This resulted in fewer observed major events than anticipated, which reduced the statistical power of the study and limited the precision of the diagnostic accuracy estimates. Furthermore, included patients more often presented high-risk characteristics, suggesting selection bias due to diagnostic suspicion. Second, the number of events was low, a common finding due to low prevalences in primary care settings, resulting in wide CIs. Third, symptoms not explicitly reported were assumed absent, which may have led to misclassification. Because this approach did not retain the distinction between ‘not documented’ and ‘documented as absent’, we were unable to quantify the amount of missing symptom information or perform sensitivity analyses based on missingness. This also affected the exploratory analyses of the updated NTS protocol, as several of its elements were not part of the original protocol or the additional study questions. Fourth, verification bias may have occurred as not all patients received diagnostic testing to confirm or exclude major events. However, our use of a delayed reference standard may have partly mitigated this. Lastly, the study was a single-centre study, which may limit generalisability.

### Comparison with literature

Safe and efficient triage of chest pain is pivotal to enable timely treatment and prevent complications of potential ACS. Prior research suggests an acceptable missed diagnosis rate of ≤1% and a maximum of 25–50 unnecessary referrals per ACS case, assuming a prevalence of 5%.^6 7 13^ These benchmarks translate into a desired NPV ≥99% and specificity ≥50%. A previous retrospective assessment of the MHS and INTERCHEST showed a potential advantage of the scores compared with the NTS protocol; however, both the scores and the NTS did not achieve the desired diagnostic accuracy.^14^ Our current prospective study confirms these results: neither score reached optimal diagnostic accuracy, and both the MHS and INTERCHEST score failed to improve triage specificity without compromising safety. For this study, we adapted the INTERCHEST score by replacing the original item ‘physician’s suspicion of a serious condition’ with the triage assistant’s sense of alarm, rated ≥5.5 on a 1–10 scale. Alternative thresholds did not significantly impact the score’s diagnostic accuracy.

In October 2024, the NTS protocol was revised to incorporate elements of the Safety First model, a clinical prediction rule developed by Wouters *et al* to predict ACS among patients with chest pain in OOH-PC.^17^ In its original form, the model showed a C-statistic of 0.77–0.79 for ACS and a more favourable diagnostic accuracy compared with the NTS protocol.^17 19^ For implementation, the model was converted to a decision tree, which had not been externally validated. In our exploratory analysis, the updated protocol achieved higher specificity but markedly reduced sensitivity, resulting in a higher number of missed major events and ACS. These findings raise safety concerns and highlight the need for further validation. A key limitation of this analysis is potential misclassification due to missing data, as several elements of the updated protocol were not part of the NTS protocol that was in use during our study.

### Clinical implications

Our findings show that neither the MHS nor the INTERCHEST score outperformed the NTS protocol, indicating that these tools may not be suitable for improving current telephone triage strategies. Although both scores include additional symptom information beyond what is used in the NTS, this added information did not translate into improved diagnostic accuracy. In addition, none of the strategies were able to significantly discriminate for ACS. Although CIs were wide, the number of events was low. The NTS protocol’s high sensitivity (97.2% for major events; 100% for ACS) is a critical strength in telephone triage, where missing urgent cases has greater clinical consequences than generating false positives. The reduced sensitivity observed with both decision rules highlights the core challenge of telephone triage: safely assessing chest pain in a setting where clinical examination and diagnostic testing are unavailable. While improved specificity could theoretically reduce unnecessary resource utilisation, the increased rate of missed urgent cases poses unacceptable safety risks.

Our study also revealed several logistical challenges. To improve feasibility, future prospective studies should aim to fully integrate study material (eg, additional triage questions) into routine workflows. If this is not feasible, a retrospective design may reduce the risk of selection bias and improve generalisability. Finally, given that our study suggests limited safety of the recently updated NTS protocol, sufficient evaluation of its performance in the near future is warranted.

### Future directions

Our findings suggest that simple clinical decision rules may not meet the demands of telephone triage for chest pain. It is also important to note that other risk-stratification tools incorporate additional clinical factors, such as point-of-care troponin testing or ECG, which may improve the diagnostic accuracy but require in-person assessment or diagnostic resources not available during telephone triage. Future research should focus on developing triage-specific tools that account for the unique constraints of telephone assessment, while maintaining high sensitivity for urgent conditions.

## Conclusions

The MHS and INTERCHEST score do not outperform current standards for telephone triage of chest pain in OOH-PC. Although both scores improved specificity, the concurrent reduction in sensitivity and increased amount of missed cases limits their clinical utility in the safety-critical context of telephone triage. The NTS protocol’s high sensitivity for detecting major events and ACS, despite lower specificity, remains appropriate for telephone triage where missing urgent cases carries greater clinical risk than generating false positives.

## Supplementary material

10.1136/bmjopen-2025-111113 online supplemental file 1

## Data Availability

Data are available on reasonable request.
