# Supplementary material for: Marburg Heart Score and INTERCHEST score for telephone triage of acute chest pain: a prospective, diagnostic accuracy study in out-of-hours primary care
Source: BMJ Open. 2026 Apr 7;16(4):e111113. doi: 10.1136/bmjopen-2025-111113 (PMC13064206; doi:10.1136/bmjopen-2025-111113)
Supplement: online supplemental file 1 [file bmjopen-16-4-s001.docx]

**Supplement 1. Additional study items**

| Upon contact with OOH-PC with key complaint of chest pain or when NTS protocol 'thoracic pain' is enabled: |
| --- |
| Inform patient on study and request verbal consent |
| **Additional triage questions:**   1. Pain reproducible with palpation? 2. Pain related to or worsened by exertion? 3. Medical history of cardiovascular disease^a^? If yes, specify. 4. Patient believes pain is of cardiac origin? |
| **Additional assessments:**   1. Triage assistant’s suspicion of a cardiac cause on a 1-10 scale? 2. Triage assistant agrees with urgency code generated by NTS protocol? |

*Supplement 1. Additional study items*
The table shows the composition of the study material used by the triage assistants to include eligible patients. After informing the patient about the study and requesting an initial verbal consent, triage assistants asked 4 additional questions and made 2 additional assessments.
Annotations: ^a^ cardiovascular disease was defined as known coronary artery disease, cerebrovascular disease, or peripheral vascular disease.
Abbreviations: out-of-hours primary care (OOH-PC); Netherlands Triage Standard (NTS).

**Supplement 2. Definition of major and non-major events**

|  | | **Final diagnosis** | **Precondition** |
| --- | --- | --- | --- |
| **Major events** | | | |
|  | *Cardiovascular conditions* | Death from any cause |  |
|  | | Acute coronary syndrome |  |
|  | | Urgent coronary revascularization |  |
|  | | Pulmonary embolism |  |
|  | | Thoracic aortic aneurysm (dissection or ruptured) |  |
|  | | Severe/Acute congestive heart failure | Hospitalization |
|  | | Severe peri(myo)carditis | Hospitalization |
|  | | Symptomatic atrial fibrillation | Hospitalization (cardioversion or converted through medication) |
|  | | Aortic valve stenosis | Hospitalization |
|  | | CVA/TIA |  |
|  | *Non-cardiovascular conditions* | (Tension) pneumothorax | Hospitalization |
|  | | Severe pneumonia | Hospitalization |
|  | | Inflammatory processes such as appendicitis, pancreatitis, cholecystitis | Hospitalization |
|  | | Other, such as: exacerbation COPD or hypertensive crisis | Hospitalization |
|  | | Traumatic event (with significant impact) | Hospitalization |
| **Non-major events** | | Stable angina pectoris | Outpatient treatment |
|  | | Mild congestive heart failure | Outpatient treatment |
|  | | Mild peri(myo)carditis | Outpatient treatment |
|  | | Atrial fibrillation (recurrent / paroxysmal) | Outpatient treatment |
|  | | Hypertension | Outpatient treatment |
|  | | Mild pneumothorax | Outpatient treatment |
|  | | Mild pneumonia | Outpatient treatment |
|  | | Mild respiratory problems (such as viral infections) |  |
|  | | Gastric/esophagus problems |  |
|  | | Muscoloskeletal |  |
|  | | Traumatic (mild trauma) | Outpatient treatment |
|  | | Mental health,panic attack,anxiety disorder |  |

*Supplement 2. Definition of major and non-major events*The table specifies the distinction between major and non-major events as defined in the TRACE study. In some diagnoses the urgency is determined by preconditions. For example, severe congestive heart failure that requires hospitalization or immediate in-hospital treatment is considered a major event, whereas mild congestive heart failure that requires only outpatient treatment is not.
*Abbreviations:* cerebrovascular accident (CVA); transient ischemic attack (TIA); chronic obstructive pulmonary disease (COPD).


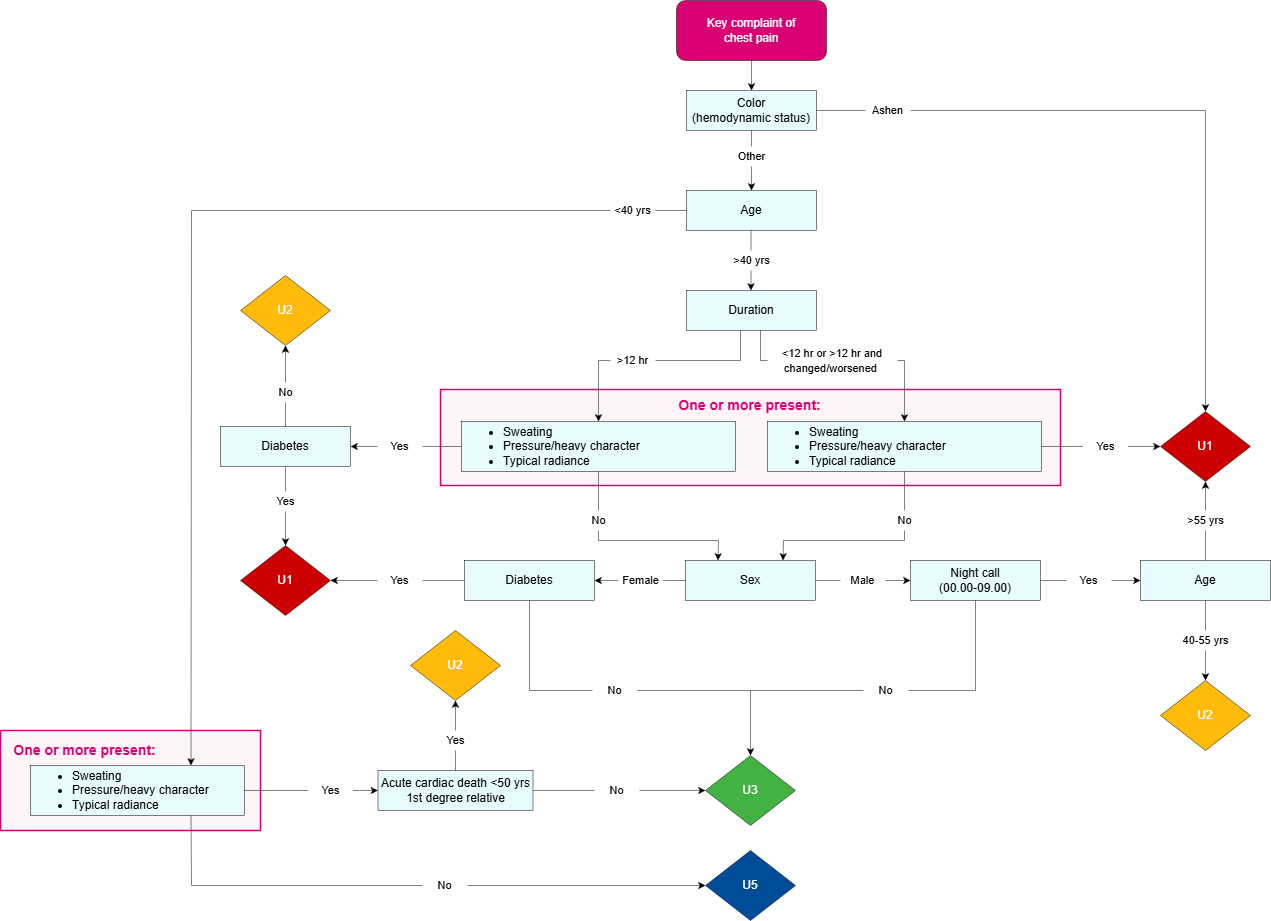
**Supplement 3. Decision tree of the updated NTS protocol (per October 2024)**

*Supplement 3. Decision tree of the updated NTS protocol (per October 2024)*
In October 2024, the NTS protocol was updated based on the Safety First model (19). Predictors from this model and elements from the prior NTS protocol were combined into a decision tree with corresponding urgency codes. Included Safety First predictors were: age, sex, sweating, and calling at night (between 00:00 and 09:00). Additionally, acute cardiac death <50 years in a first degree relative was included. Original NTS questions on pain severity, location and course were omitted. The urgency codes were based on the relative risk of an ACS or other life threatening event in the derivation cohort of the Safety First model (i.e. U1: ≥20%, U2: 15-20%, U3: 10-15%, U4; 5-10% and U5: <5%).
The decision tree displayed here was adopted and translated based on the original document: “Revision of the protocol for chest pain – supporting document and background information” released by the Julius Centrum UMC Utrecht and the Netherlands Triage Standard in September 2024 to support the alterations made to the protocol (20).

**Supplement 4. Diagnostic accuracy of the updated NTS protocol (per October 2024) for predicting major events and ACS**

|  | Threshold | Positive test | TP | FP | FN | TN | Sensitivity (%) | Specificity (%) | PPV (%) | NPV (%) |
| --- | --- | --- | --- | --- | --- | --- | --- | --- | --- | --- |
| **Major event** |  |  |  |  |  |  |  |  |  |  |
| NTS protocol | U1/U2 | 33.6% | 14 | 80 | 22 | 164 | 38.9 (23.1-56.5) | 67.2 (60.9-73.1) | 14.9 (10.1-21.5) | 88.2 (85.0-90.8) |
| **ACS** |  |  |  |  |  |  |  |  |  |  |
| NTS protocol | U1/U2 | 33.6% | 7 | 87 | 6 | 180 | 53.9 (25.1-80.8) | 67.4 (61.4-73.0) | 7.5  (4.5-12.1) | 96.8 (94.3-98.2) |

*Supplement 4. Diagnostic accuracy of the updated NTS protocol (per October 2024) for predicting major events and ACS.*
The table lists the diagnostic performance of the updated NTS protocol for chest pain, installed in October 2024.  *Abbreviations:* acute coronary syndrome (ACS), true positive (TP), false negative (FN), false positive (FP), true negative (TN), positive predictive value (PPV), negative predictive value (NPV), Netherlands Triage Standard (NTS).
